# Integrative analysis of expression, prognostic significance and immune infiltration of RFC family genes in human sarcoma

**DOI:** 10.18632/aging.204039

**Published:** 2022-04-29

**Authors:** Gen Wu, Jian Zhou, Xi Zhu, Xianzhe Tang, Jie Liu, Qiong Zhou, Ziyuan Chen, Tang Liu, Wanchun Wang, Xungang Xiao, Tong Wu

**Affiliations:** 1 Department of Orthopedics, The Second Xiangya Hospital of Central South University, Changsha 410011, Hunan, China; 2 Clinical Medicine Eight-Year Program, Central South University, Changsha 410013, Hunan Province, China; 3 Department of Internal Medicine III, University Hospital, Ludwig Maximilian University, Munich 81377, Germany; 4 Department of Orthopedics, Chenzhou No.1 People's Hospital, Chenzhou 423000, Hunan, China; 5 Department of Cardiology, The Fourth Hospital of Changsha, Changsha 410006, Hunan, China; 6 Department of Orthopedics, The First People's Hospital of Changde City, Changde 415003, Hunan, China; 7 Department of Emergency, The First Hospital of Changsha, Changsha 410005, Hunan, China

**Keywords:** RFC, prognosis, expression, KEGG, bioinformatics analysis

## Abstract

Objective: To reveal the expression and prognostic value of replication factor C family genes (RFCs) in patients with sarcoma.

Results: The results showed that the mRNA expression levels of RFC2, RFC3, RFC4, and RFC5 were increased in sarcoma tissues. In addition, Cancer Cell Line Encyclopedia (CCLE) dataset analysis indicated that RFC1, RFC2, RFC3, RFC4, and RFC5 were elevated expressed in sarcoma cell lines. Moreover, Gene Expression Profiling Interactive Analysis (GEPIA) and Kaplan-Meier Plotter showed that highly expressed RFC2-5 were associated with poor overall survival (OS) or relapse-free survival (RFS) in sarcoma patients. The results of the Tumor Immune Estimation Resource (TIMER) database indicated that the expression of RFCs was negatively correlated with the infiltration of CD4+ T cells and macrophages.

Conclusions: There were significant differences in the expression of RFCs between normal tissue and sarcoma tissue, and RFC2, RFC3, RFC4, and RFC5 might be promising prognostic biomarkers for sarcoma.

Methods: The expression of RFCs was analyzed using the ONCOMINE dataset and GEPIA dataset. CCLE dataset was used to assess the expression of RFCs in the cancer cell line. The prognostic value of RFCs was evaluated by GEPIA and Kaplan-Meier analysis. Furthermore, the association between RFCs and their co-expressed genes were explored via ONCOMINE and GEPIA datasets. We used the TIMER dataset to analyze the immune cell infiltration of RFCs in sarcoma.

## INTRODUCTION

The replication factor C (RFC, activator 1) was first purified from the extracts of HeLa cells in human cervical cancer, participates as an important host factor in the replication of DNA [1, 2]. As a primer identification factor for DNA polymerase, RFC is a DNA binding protein with a specific structure and function [3]. *In vivo*, RFC plays an essential in cell biology cycles as a regulatory protein [4]. In humans, RFC is reported as a complex consisting of RFC1 (140 kDa), RFC2 (40 kDa), RFC3 (38 kDa), RFC4 (37 kDa) and RFC5 (36 kDa) subunits [5]. The binding of the five subunits determines the physiological function of RFC. According to reports [6, 7], RFC can participate in excision repair and mismatch repair of damaged DNA by initiating signal transduction downstream of the checkpoint at the site of DNA damage by binding to the cell cycle checkpoint protein. In addition, RFC can load DNA polymerase and proliferating cell nuclear antigen (PCNA) onto the primer-bound DNA template to form a DNA-RFC-PCNA-DNA polymerase complex. And then, the polymerase complex extended along with the DNA template in the presence of deoxynucleotides (dNTPs), via the action of human single-stranded DNA binding protein (hSSB) [4]. As for interacting partners with a variety of proteins, not only are RFC factors involved in multiple processes in the normal cell cycle, but RFC factors also play an essential role in the transcription and proliferation of tumor cells.

Further studies indicated that in the RFC family, different subunits have different roles in the cell cycle [4]. RFC1 DNA-binding domain contains the main, and of PCNA interacts directly with, involved in DNA synthesis, DNA repair, and cell cycle. Unlike other subunits, RFC1 is rarely reported to have a relationship with sarcoma. In the studies of Tang [8] and Pennaneach [9], it is pointed out that RFC1 can promote cell survival after DNA damage through the retinoblastoma (Rb) pathway, which is related to Hutchinson-Gilford Progeria Syndrome (HGPS). According to reports [10], RFC2 is one of the important components of the RFC complex that can unload PCNA and inhibit DNA polymerase activity, it is highly expressed in some sarcoma tissues and cells. RFC2, as a key gene, was upregulated in metastatic samples from Ewing's sarcoma patients [11]. Meanwhile, bioinformatics analysis showed that the up regulation of this key gene reduced the overall survival rate of Ewing's sarcoma patients. As the dominant gene in the 13q13 amplicon, RFC3 is considered to be an oncogene or anti-oncogene in different cancers based on its cellular and histological characteristics [12]. Recently, the study suggested that RFC3 is regulated by a series of miRNAs including miR-802 [13]. At the same time, it is reported that the up-regulated expression of miR-802 is shown in osteosarcoma tissues and promotes cell proliferation by targeting p27 in U27 OS and MG-63 cells [14]. Hence, RFC3 is also closely related to the cell proliferation of sarcoma tissue. In the DNA damage checkpoint pathway, RFC4 plays an important role and can enhance the anti-tumor activity of DNA-damaging chemotherapeutics [15]. A study has pointed out that changes in cell cycle regulation occur in several types of cancer, including osteosarcoma [16]. RFC4 interacts with CDK1, MAD2L1, NDC80, and BUB1, and acts on cell mitosis and cell cycle [13]. RFC5 is a necessary subunit to open the PCNA clamp during DNA replication. RFC5 participates in the repair and regulation of mismatches, nucleotide excision, cell cycle, and DNA double helix damage, [17, 18]. Studies have suggested that RFC5 is significantly up-regulated in various cancer tissues or cells, and its expression increases as the disease progress [19–22]. However, the specific role of RFC5 in sarcoma is rarely expressed in more detail. So far, the expression program, functional role in sarcoma tissues, and impact on the prognosis of sarcoma patients by RFC5 are still poorly known.

In sarcoma patients, the pathological features conferred by RFC with different expression levels and their prognostic impact in these patients have been reported [4, 13]. To the best of our knowledge, there is still no research using bioinformatics to analyze the role of the RFC family in sarcoma. In our study, we summarized the expression and mutations of RFC genes in sarcoma to further analyze their process, latent function, and prognosis of sarcoma transcription levels.

## RESULTS

### The transcription level of RFCs in patients with sarcoma

**Figure 1:**
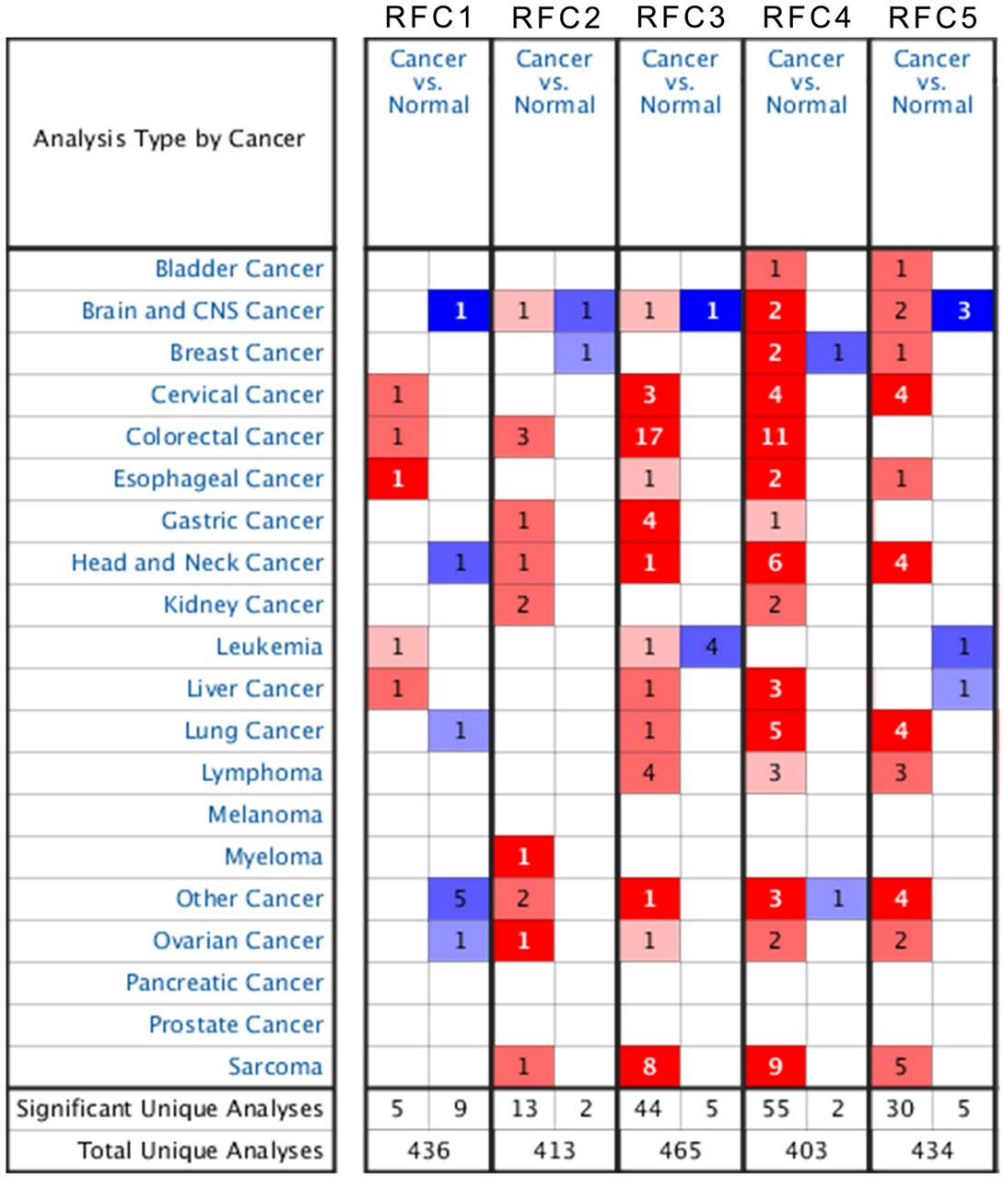
The transcription level of RFCs in patients with sarcoma.

In mammalian cells, there have been identified five kinds of RFC factors. In the ONCOMINE database, the transcription level of RFC in cancer tissues was different from that in normal tissues (Figure 1). The mRNA transcription level of RFCs showed a significant difference between normal and sarcoma patients, except RFC1. In Detwiller Sarcoma’s dataset [23], RFC2, with a fold change of 3.287, was overexpressed in Fibrosarcoma (Table 1). In the database of Detwiller sarcoma [23], RFC3 expressed an increase in fibroids with a multiple change of 3.184. Detwiller sarcoma’s dataset [23] showed that RFC3 expression factor with the increased expression: the change of RFC3 in Round Cell Liposarcoma was 3.588, the change of RFC3 in patients with Synovial Sarcoma was 2.548, and the change of patients with Leiomyosarcoma was 2.624 (Table 1). In Barretina Sarcoma’s dataset [24], RFC3 was over-expressed than normal in the following sarcomas: 2.413 in myxoid/round cell liposarcoma, 2.257 in myxofibrosarcoma, 2.514 in leiomyosarcoma, and 2.539 in pleomorphic liposarcoma.

**Table 1:** The significant changes of rfc expression in transcription level between different types of sarcoma, NA: not available.

**Gene ID**	**Types of sarcoma vs. normal**	**Fold change**	**P value**	**t test**	**Renferences**
**RFC1**	NA	NA	NA	NA	NA
**RFC2**	Fibrosarcoma vs. Normal	3.287	6.42E-5	4.779	Detwiller Sarcoma
**RFC3**	Fibrosarcoma vs. Normal	3.184	5.00E-7	7.154	Detwiller Sarcoma
	Round Cell Liposarcoma vs. Normal	3.588	6.65E-7	7.393	Detwiller Sarcoma
	Synovial Sarcoma vs. Normal	2.548	6.13E-6	6.366	Detwiller Sarcoma
	Leiomyosarcoma vs. Normal	2.624	5.72E-5	5.351	Detwiller Sarcoma
	Myxoid/Round Cell Liposarcoma vs. Normal	2.413	5.49E-13	12.673	Barretina Sarcoma
	Myxofibrosarcoma vs. Normal	2.257	3.37E-11	9.037	Barretina Sarcoma
	Leiomyosarcoma vs. Normal	2.514	6.20E-10	8.700	Barretina Sarcoma
	Pleomorphic Liposarcoma vs. Normal	2.539	9.98E-8	7.117	Barretina Sarcoma
**RFC4**	Leiomyosarcoma vs. Normal	7.003	1.06E-9	10.790	Detwiller Sarcoma
	Pleomorphic Liposarcoma vs. Normal	3.658	4.03E-7	7.948	Detwiller Sarcoma
	Malignant Fibrous Histiocytoma vs. Normal	4.337	1.15E-7	7.444	Detwiller Sarcoma
	Fibrosarcoma vs. Normal	3.579	6.63E-7	6.866	Detwiller Sarcoma
	Leiomyosarcoma vs. Normal	7.827	1.46E-17	16.192	Barretina Sarcoma
	Pleomorphic Liposarcoma vs. Normal	4.682	4.85E-15	14.216	Barretina Sarcoma
	Myxofibrosarcoma vs. Normal	4.518	1.33E-15	17.566	Barretina Sarcoma
	Myxoid/Round Cell Liposarcoma vs. Normal	3.952	5.27E-12	18.791	Barretina Sarcoma
	Dedifferentiated Liposarcoma vs. Normal	3.099	3.56E-12	14.411	Barretina Sarcoma
**RFC5**	Myxofibrosarcoma vs. Normal	2.003	2.59E-13	10.719	Barretina Sarcoma
	Pleomorphic Liposarcoma vs. Normal	2.097	7.02E-9	7.985	Barretina Sarcoma
	Leiomyosarcoma vs. Normal	5.371	7.61E-6	6.193	Detwiller Sarcoma
	Fibrosarcoma vs. Normal	3.255	1.73E-5	5.368	Detwiller Sarcoma
	Malignant Fibrous Histiocytoma vs. Normal	4.134	8.80E-5	4.686	Detwiller Sarcoma

Detwiller Sarcoma’s dataset [23] suggests that RFC4 over-expression was found in Leiomyosarcoma with a fold change of 7.003, RFC4 over-expression was found in Pleomorphic Liposarcoma with a fold change of 3.658, RFC4 over-expression was found in Malignant Fibrous Histiocytoma with a fold change of 4.337, and RFC4 over-expression was found in Fibrosarcoma with of a fold change of 3.579. In Barretina Sarcoma’s dataset [24], RFC4 was overexpressed in Leiomyosarcoma with a fold change of 7.827. Barretina Sarcoma’s dataset [24] also indicated that RFC4 overexpression is found in Pleomorphic Liposarcoma with a fold change of 4.682. RFC4 over-expression was found in Myxofibrosarcoma with a fold change of 4.518, in Myxoid/Round Cell Liposarcoma with a change of 3.952, and in Dedifferentiated Liposarcoma with a change of 3.099.

In the 2 databases, there were significant differences in mRNA transcription levels of RFC5. In Barretina Sarcoma’s dataset [24], RFC5 over-expression was found in Myxofibrosarcoma with a change of 2.033 compared with normal, and in Pleomorphic Liposarcoma with a change of 2.097. In Detwiller Sarcoma’s dataset [23], RFC5 over-expression was found in Leiomyosarcoma with a fold change of 5.371, in Fibrosarcoma with a change of 3.255, and in Malignant Fibrous Histiocytoma with a change of 4.134.

### Relationship between the mRNA transcription levels and the clinical pathological parameters in RFC in sarcoma patients

**Figure 2:**
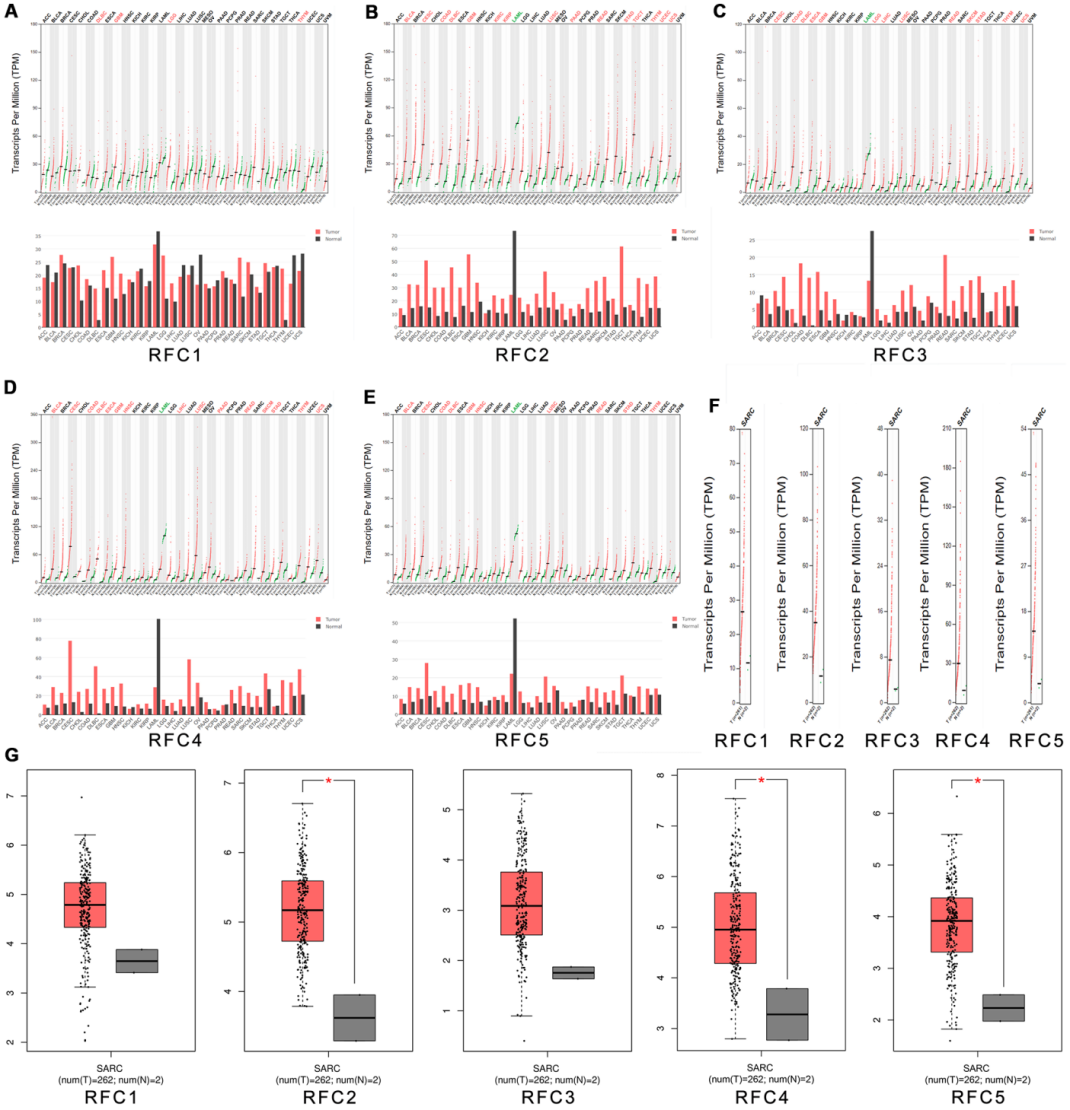
**The expression of RFCs in sarcoma.** (**A**) The expression of RFC1 in pan-cancer. (**B**) The expression of RFC2 in pan-cancer. (**C**) The expression of RFC3 in pan-cancer. (**D**) The expression of RFC4 in pan-cancer. (**E**) The expression of RFC5 in pan-cancer. (**F**, **G**) The expression of RFCs in SARC.

We use the Gene Expression Profiling Interactive Analysis (GEPIA) dataset (http://gepia.cancer-pku.cn/) to compare different mRNA expression levels of RFCs in sarcoma and normal samples. The results showed that RFC2, RFC4, and RFC5 were upregulated in sarcoma patients, while the high expression levels of RFC1 and RFC3 were both with no significance. (Figure 2A–2G).

### Expression of RFC transforming factors in sarcoma cell lines

**Figure 3:**
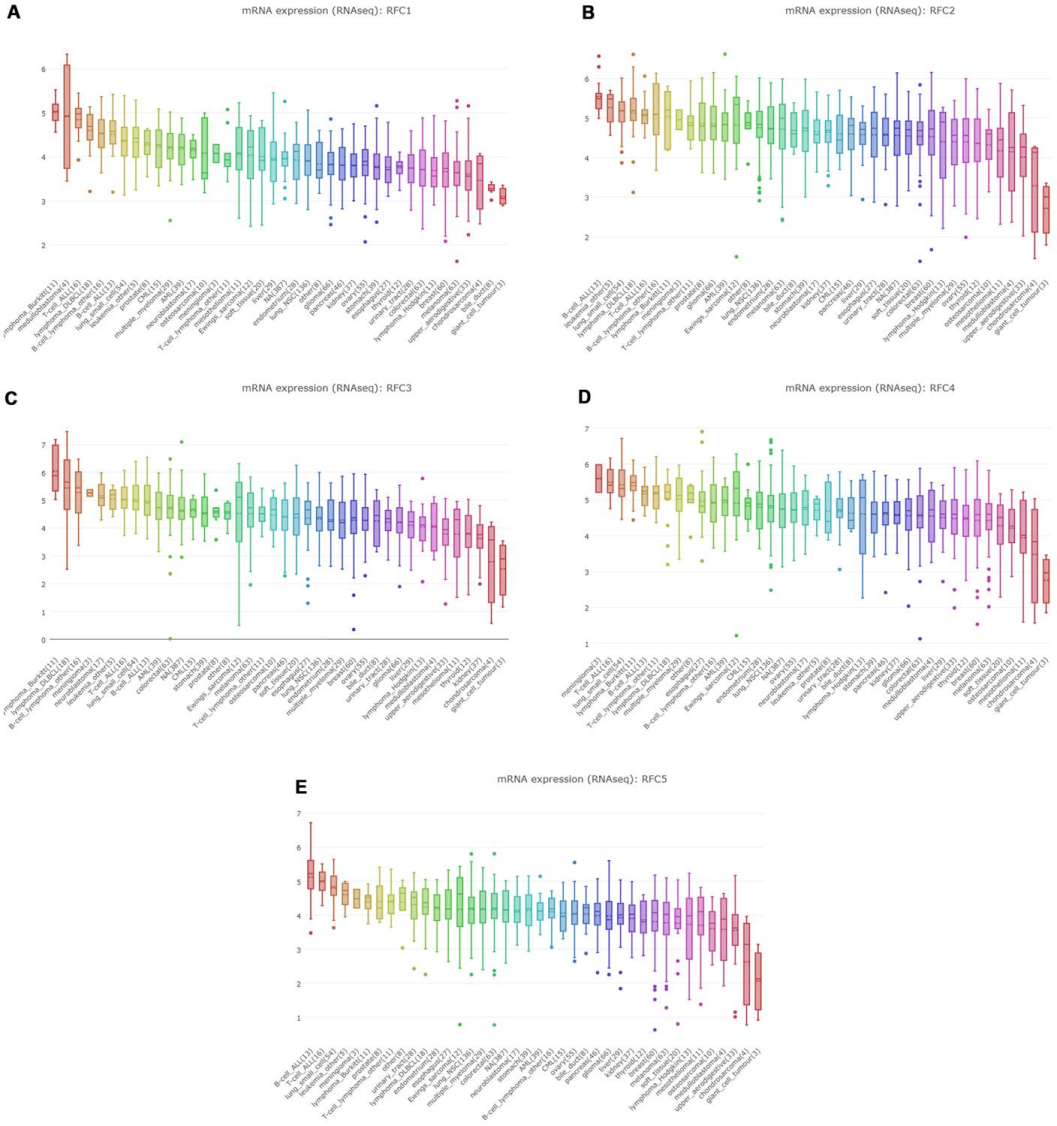
**The expression of RFCs in sarcoma cell lines.** (**A**) The expression of RFC1 in sarcoma cell lines, analyzed by CCLE. (**B**) The expression of RFC2 in sarcoma cell lines, analyzed by CCLE. (**C**) The expression of RFC3 in sarcoma cell lines, analyzed by CCLE. (**D**) The expression of RFC4 in sarcoma cell lines, analyzed by CCLE. (**E**) The expression of RFC5 in sarcoma cell lines, analyzed by CCLE.

Through the Cancer Cell Line Encyclopedia, we expanded our preclinical human cancer model of detailed annotation process (https://www.broadinstitute.org/ccle). The expressions of RFC1-5 were high in sarcoma cell lines (Figures 3A–3E).

### Prognostic value of RFCs in sarcoma

**Figure 4:**
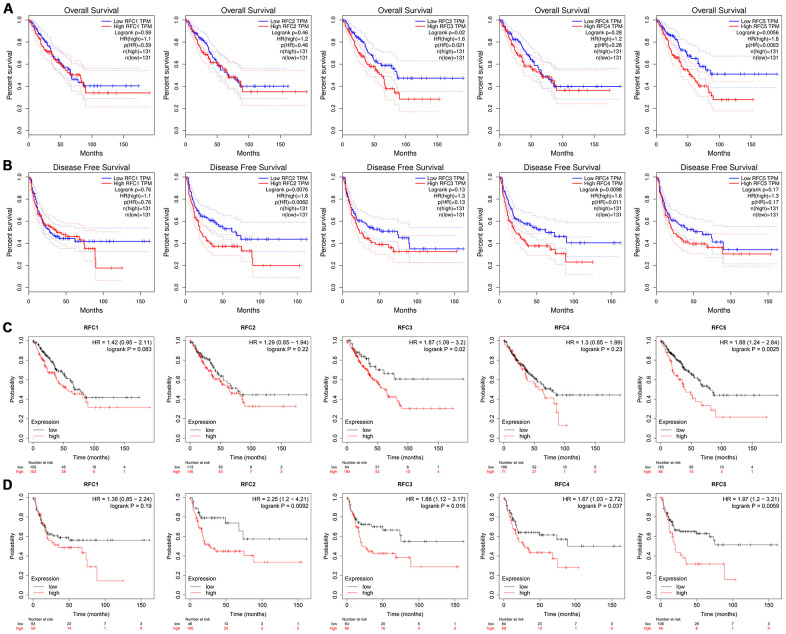
**The prognostic value of mRNA level of RFC factors in sarcoma patients.** (**A**, **B**) The prognostic value of mRNA level of RFC factors in sarcoma patients, analyzed by GEPIA. (**C**, **D**) The prognostic value of mRNA level of RFC factors in sarcoma patients, analyzed by Kaplan-Meier plotter.

We investigated the prognostic analysis of RFC1-5 in sarcoma using the plotter tool in the GEPIA and Kaplan Meier databases (Kaplan Meier plotter). Interestingly, in these two databases, poor overall survival (OS) and disease-free survival (DFS) of sarcoma were related to the upregulation of RFC1, but with meaningless (Figure 4). The results, however, of the database suggested that high expression of RFC2 and RFC4 were associated with the poor DFS and RFS in sarcoma (Figure 4B, 4D), with statistical differences. Nevertheless, increased RFC3 and RFC5 mRNA levels were associated with poor OS and RFS in sarcoma (Figure 4A, 4C, 4D).

### Co-expressed RFC genes and the correction between RFCs in sarcoma

**Figure 5:**
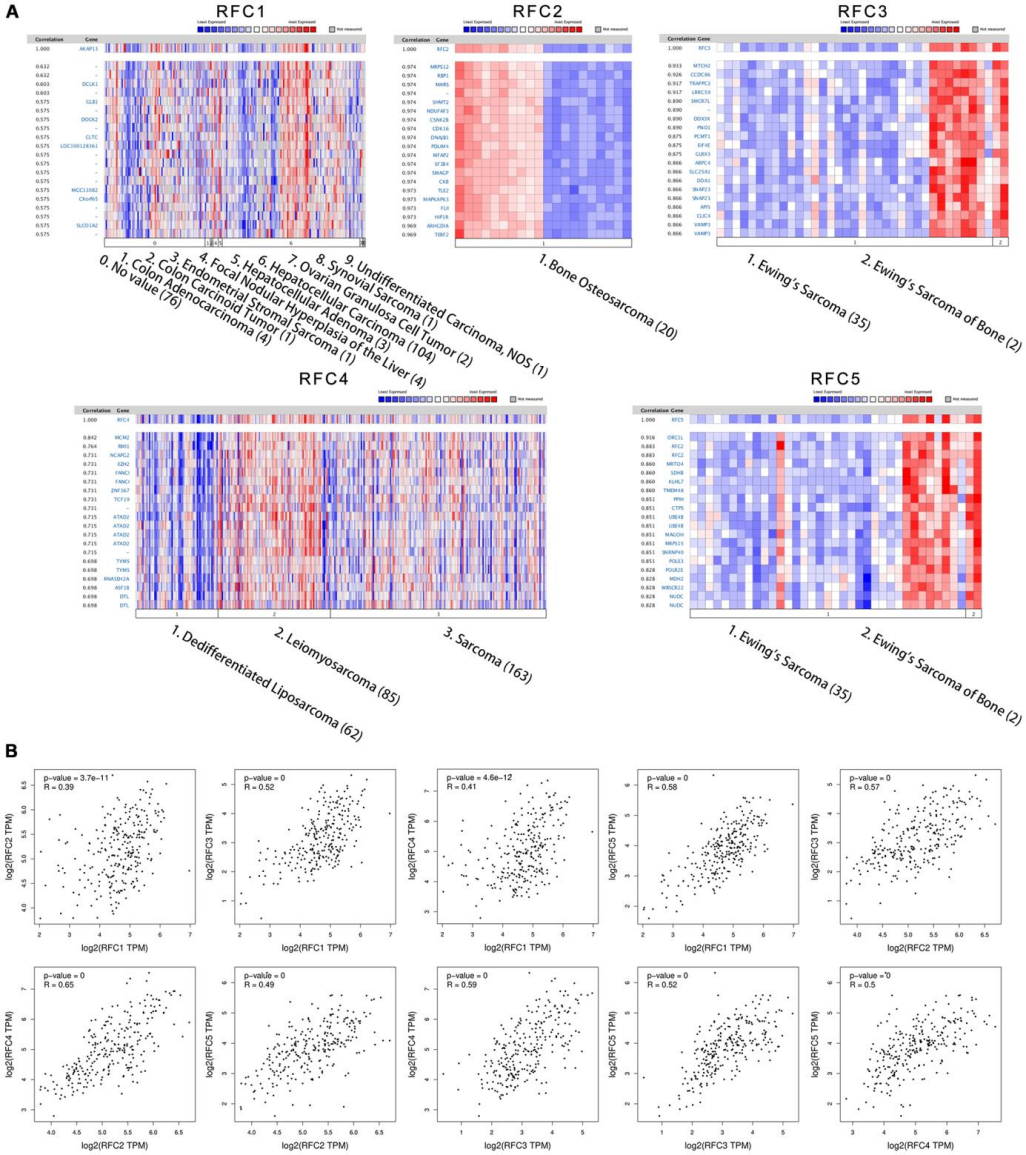
**Co-expression genes of RFCs, and the correction between RFCs in sarcoma.** (**A**) Co-expression genes of RFCs in sarcoma, analyzed by Oncomine. (**B**) The correction between RFCs in sarcoma, analyzed by GEPIA.

Analyzed genes co-expressed with RFC1, in Chen’s study [25], we found that RFC1 has been positively corrected by AKAP13, DCLK1, GLB1, DOCK2, CLTC, LOC100128361, MGC11082, CXorf65, and SLCO1A2. And then we analyzed genes co-expressed with RFC2 in the study of Stossi [26], the results showed that RFC2 has been positively corrected by MRPS12, RBP1, MARS, SHMT2, NDUFAF3, CSNK2B, CDK16, DNAJB1, PDLIM4, MFAP2, SF3B4, SMAGP, CKB, TLE2, MAPKAPK3, FLII, HIP1R, ARHGDIA, and TERF2. Analyzed genes co-expressed with RFC3 in the study of Schaefer [27], we found that RFC3 has been positively corrected by MTCH2, CCDC86, TRAPPC3, LRRC59, SMCR7L, DDX3X, PNO1, PCMT1, EIF4E, GLRX3, ARPC4, SLC25A1, DDA1, SNAP23, API5, CLIC4, and VAMP3. Genes co-expressed with RFC4 were described in Chibon’s study [28], the results showed that RFC4 was positively corrected with MCM2, RMI1, NCAPG2, EZH2, FANCI, ZNF367, ATAD2, TYMS, RNASEH2A, ASF1B, and DTL. Genes co-expressed with RFC5 in the study of Chen [25], and we found RFC5 was positively corrected with ORC1L, RFC2, MRTO4, SDHIB, TMEM48, PPIH, CTPS, UBE4B, MAGOH, MRPS15, SNRNP40, POLE3, MDH2, WBSCR22, and NUDC (Figure 5A). Through the GEPIA database, we analyzed the mRNA expressions and calculated the correlations between RFCs with each other. The results showed that RFC1 was positively corrected by RFC2 (R=0.39, p<0.05), RFC3 (R=0.52, p<0.05), RFC4 (R=0.41, p<0.05), and RFC5 (R=0.58, p<0.05) (Figure 5B). Furthermore, RFC2 was positively corrected with RFC3(R=0.57, p<0.05), RFC4(R=0.65, p<0.05), and RFC5(R=0.49, p<0.05) (Figure 5B). RFC3 was both positively corrected by RFC4(R=0.59, p<0.05), and RFC5(R=0.52, p<0.05). RFC4 was positively corrected by RFC5(R=0.5, p<0.05) (Figure 5B).

### Association between RFCs and infiltration levels of immune cells in sarcoma

**Figure 6:**
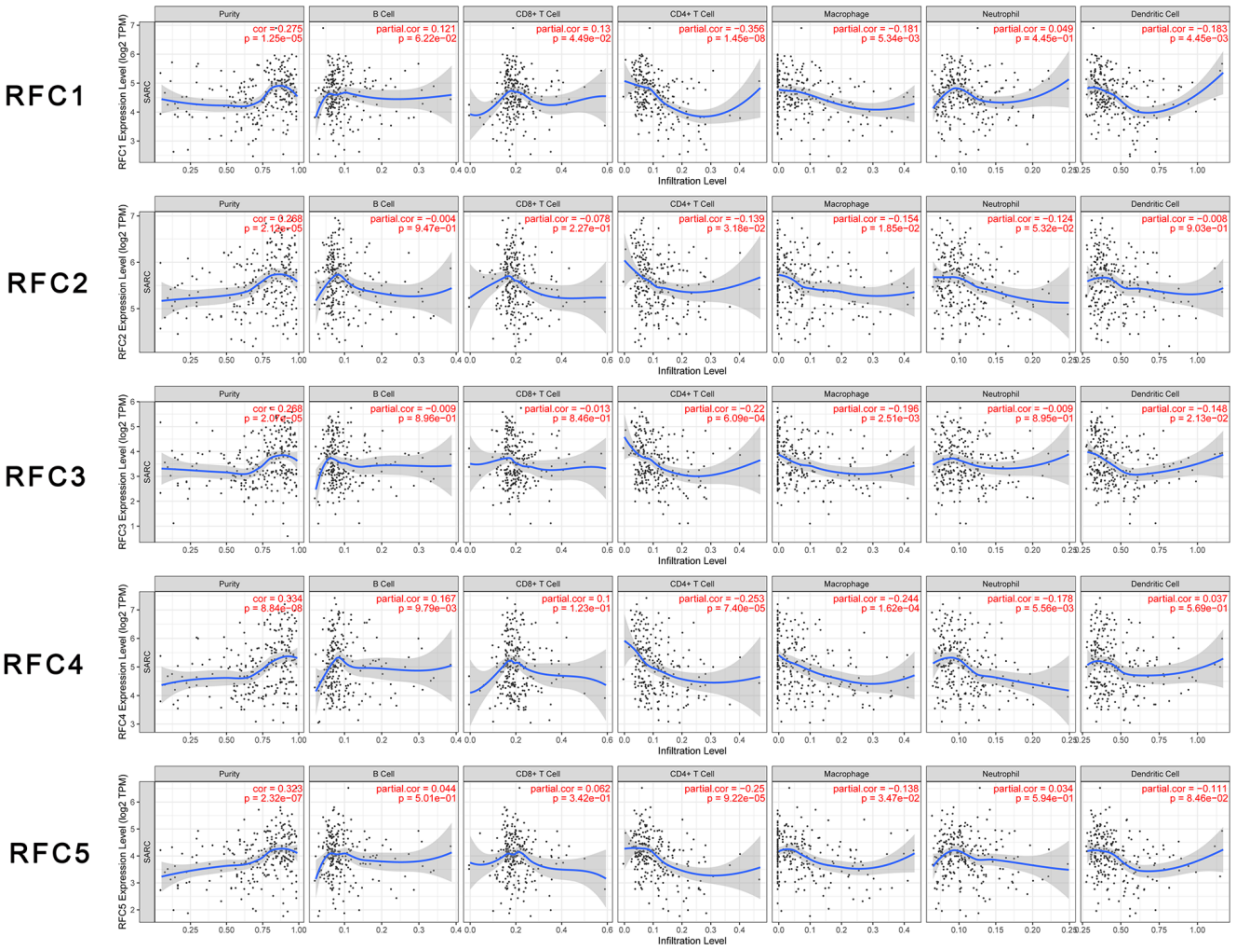
**Relationship between differentially expressed RFC genes and immune cell infiltration.** The immune cells we analyzed included B cells, CD8+ T cells, CD4+ T cells, macrophages, neutrophils, and dendritic cells.

We explored the relationship between the expression level of RFCs and immune cell infiltration in sarcoma using the TIMER database. The results showed that RFC1-5 were all positively correlated with tumor purity, negatively correlated with the infiltration of CD4+ T cell and macrophage, with significance. (Figure 6).

## DISCUSSION

The role of RFC factor dysregulation in many types of cancer has been reported [29–32]. Similarly, we found that mRNA expression levels of RFC2, RFC3, RFC4, and RFC5 from RFC family genes were upregulated in sarcoma tissues. The imbalance of RFC may lead to the disorder of the cell cycle, which is no longer regulated by normal physiological mechanisms, and may further lead to the cancerization of normal tissues. RFC1 is generally considered to be an important part of DNA replication and repair in the RFC family [33]. Moggs et al. [34] found that when the expression of RFC1 is inhibited, the proliferation of estrogen receptor-negative breast cancer cells is also inhibited. Bermudez et al. [35] pointed out that overexpression of RFC1 can prevent histone H3K56 from over-acetylation. Celic et al. [36] indicated that over acetylated histone H3K56 will induce cell death. Through our study, although in ONCOMINE database suggested that the mRNA expression of RFC1 gene in sarcoma patients was non-difference from the normal, CCLE database indicated that that the RFC1 gene expression in sarcoma cell lines shows a high expression status, we can still consider that RFC1 gene is highly expressed in sarcoma cells. Using the GEPIA and Kaplan Meier plotter databases, we determined the prognostic value of the RFC1 gene in sarcoma, and although high expression of RFC1 was associated with poor OS and DFS in sarcoma patients, it was not statistically significant, so RFC1 expression did not predict sarcoma patients related prognosis.

Some evidence suggested that RFC2 was a key gene, and its upregulation was related to the metastasis and prognosis of Ewing’s sarcoma [11]. Xiong et al. [37] showed that RFC2 was closely related to nasopharyngeal carcinoma, and the up regulation of RFC2 expression was obviously high in patients with nasopharyngeal carcinoma than normal. Meanwhile, other studies have shown that the expression level of RFC2 was also higher in the normal tissues than in patients with glioblastoma [38]. There was a study pointed out that in patients with choriocarcinoma, high expression of RFC2 may have the effect of predicting the prognosis of the disease [39]. Therefore, RFC2 may play an oncogene in a variety of malignant tumors. Until now, there is no definite research showing the specific effect of RFC2 in sarcoma. In our study, the expression of RFC2 in fibrosarcoma was 3.287 times higher than in normal tissues. Through the CCLE database, we found that RFC2 was also up-regulated in sarcoma cell lines. Meanwhile, in the analysis of GEPIA and Kaplan Meier plotter, we also found that patients with sarcoma with high expression of RFC2 had poor disease-free survival (DFS), which was statistically significant. Therefore, RFC2 not only plays an oncogene role in fibrosarcoma but also has a latent function in predicting disease outcomes in sarcoma.

In a study on osteosarcoma [13], it was pointed out that RFC3, CDK1, MAD2L1, NDC80, BUB1, etc. jointly participated in the related links of the disease prognosis of osteosarcoma. According to the study of patients with acute myeloid leukemia (AML) [40], RFC3 was found to be highly expressed in their tumor cells. In addition, in the study of ovarian cancer patients [12], the average survival time of patients with high RFC3 expression levels was only 7.7 months, while that of patients with normal expression was as long as 92.9 months. Highly expressed RFC3 has been confirmed to be a predictive gene in Kaposi’s sarcoma [41], breast cancer [42], esophageal adenocarcinoma [43], and hepatocellular carcinoma [44]. In summary, this means that RFC3 may act as an oncogene in cancers. Consistent with our analysis, in this study, the RFC3 high expression was found in sarcoma tissue. Using the CCLE database, the results suggested that RFC3 was also highly expressed in human sarcoma. In ONCOMINE, the mRNA expression level of RFC3 was up-regulated, which was specifically reflected in fibrosarcoma, leiomyosarcoma, mucosal fibrosarcoma, smooth muscle sarcoma, pleomorphic liposarcoma, and so on. Meanwhile, the results illustrated that highly expression RFC3 was associated with poor OS in patients with sarcoma, which was statistically significant.

Previous study has shown that RFC4 is highly expressed in cancer tissues such as hepatocellular carcinoma [15], non-small cell lung cancer (NSCLC) [45], prostate cancer [46], breast cancer [20], and cervical cancer [47]. In addition, some scholars further proposed that RFC4 may serve as a potential prognostic biomarker and therapeutic target [4]. In general, RFC4 has been discussed and studied in various cancers, but its exact function in sarcoma has not been described yet. At the same time, highly expressed RFC4 was related to the RFS difference in sarcoma, and there was statistical significance, that is, the DFS of sarcoma patients with high expression of RFC4 was worse. It seemed that RFC4 not only acted as an oncogene in sarcoma patients but also had a certain predictive effect on the prognosis of sarcoma patients.

RFC5 can repair mismatches, DNA double helix damage, and nucleotide excision during the cell cycle [18]. These biological characteristics have been confirmed in research to be related to the progression of cancer [17]. Similar to other subunits of the RFC family, RFC5 is expressed in a variety of cancers, such as head and neck squamous cell carcinoma [48], prostate cancer [49], cervical cancer [50], and diffuse large B-cell lymphoma (DLBCL) [51] than the normal. Hence the exact function of RFC5 in sarcoma is still inconclusive. In our study, highly expressed RFC5 was significantly correlated with poor OS in sarcoma patients, and there was statistical significance. It suggests that patients with high expression of rfc5 sarcoma usually have poor overall survival.

The expression levels of the five RFC family members were negatively correlated with the infiltration of CD4+ T cells and macrophages. Immune cells in the tumor microenvironment can promote or inhibit tumor activity, which is considered to be an important determinant of clinical outcomes and immunotherapy. The RFCs expression levels were negatively correlated with the infiltration level of CD4+ T cells and macrophages. Past studies have shown that CD4+ T cells played active roles in anti-tumor immunity. CD4+ T cells could target tumor cells in a variety of ways, directly eliminate tumor cells through the cytolytic mechanism, or indirectly interact with tumor cells by regulating the tumor immune microenvironment [52]. This indicated that the expression of RFCs might affect the development of sarcoma by regulating the infiltration of immune cells, thereby affecting the prognosis of patients. This study could provide more detailed immune information for the immunotherapy of sarcoma patients.

After understanding the heterogeneity and complexity of the molecular biology of sarcoma, this study systematically analyzed the expression and predictive value of RFC in sarcoma. Our results indicated that increased expression of RFC2-5 in sarcoma tissue might play an important role in the occurrence of sarcoma and could be used as a potential indicator of diagnosis. The expression level of RFC2-5 has predictive effects on the survival period of patients with sarcoma. Thus, transcriptional RFC2-5 are latent prognostic markers to improve the survival and prognostic accuracy of sarcoma.

However, such a study still has some limitations. First, it can only respond to some relationships between RFC factors and sarcomas, and the role of some subunits in sarcomas under a partially defined pathological classification, so it cannot make a deeper analysis. Second, disadvantages still exist in the precision of treatment and prediction, but some genomic, as well as proteomic evidence, can be provided for the study of the corresponding sarcomas. Further studies also need to combine immunohistochemical as well as correlative analysis of tumor cytology, which will facilitate the diagnosis and treatment of sarcomas with greater precision. Third, the ONCOMINE database had been taken offline on January 17, 2022, so the figure with case numbers in specific subtype vs ‘normal’ cases cannot be obtained.

## CONCLUSIONS

In conclusion, this finding systematically showed the expression of the RFC gene in sarcoma tissue and the prognosis effect on sarcoma patients. Our results indicate that increased expression of RFC2-5 in sarcoma tissue may show an essential role in the occurrence of sarcoma and can be used as latent indicators for diagnosing sarcoma. Expression levels of RFC2-5 have a predictive effect on the total survival (OS) or disease-free survival (DFS) of sarcoma patients. Therefore, transcription of RFC2-5 is a potential prognostic marker for improving survival and prognostic accuracy in sarcoma patients. The results of this study may provide new ideas for diagnosis and prognosis in sarcoma patients to select potential prognostic biomarkers.

## MATERIALS AND METHODS

### ONCOMINE analysis

ONCOMINE (https://www.oncomine.com/) is an online microarray database focused on cancer. In different cancers, it can analyze the transcript levels of the RFC gene family. Comparison of mRNA expression of the RFC gene family in clinical cancer tissue specimens and mRNA expression in normal controls was performed using the student's t-test for significance of the mean difference. Respectively, the cut-off was defined as 0.01, and the fold change of p-value was 2.

### GEPIA dataset

Gene expression analysis interactive analysis dataset (GEPIA), a newly developed interactive online gene bioinformatics analysis platform, is a way of RNA sequencing analysis. The database contains data from 9736 tumor tissues and 8587 normal tissues from The Cancer Genome Atlas (TCGA) as well as Genotype-Tissue Expression (GTEx) (http://gepia.cancer-pku.cn/) GEPIA shows a series of custom-made features such as gene differential expression analysis of tumor tissue versus normal tissue, but also correlation analysis depending on the type of pathological stage of cancer, analysis of patient survival, similar genetic tests, correlation analysis, and dimension reduction analysis.

### CCLE dataset

CCLE (Cancer Cell Line Encyclopedia), an oncogenomics research project, is led by the Broad Institute of MIT at Harvard. Similar to the above databases, CCLE is also a large, publicly available tumor genome database. It collects and collates profiling data of 1457 tumor cell lines, 84434 genomes, which includes gene expression data, chromosomal copy data as well as massively parallel sequencing data. In sarcoma cell lines, we illustrated the expression levels of the RFC gene family using the CCLE database.

### Kaplan-Meier plotter

The Kaplan Meier plotter is a publicly available online database (https://www.kmplot.com) constructed based on microarray and RNA SEQ data from public databases such as TCGA, geo, and EGA. The prognostic value of signal transducer and activator of transcription protein (STAT), as well as mRNA expression, was assessed using this online database containing expression data and survival information for the analyzed genes as well as 259 clinical sarcoma patients. To analyze the overall survival (OS) and progression-free survival (RFS), of patients with sarcoma, those samples were divided into two groups by median expression (high expression vs. low expression) and evaluated by Kaplan Meier survival plots with hazard ratios (HR, hazard ratio) with 95% confidence intervals (CI, confidence interval) and log-rank P values. TIMER database Through the TIMER database, we evaluated the effect of RFC expression level on tumor immune cell infiltration. TIMER database contains the data of tumor-infiltrating immune cells in more than 10000 samples of 32 types of cancers from TCGA [53]. On this basis, we analyzed the effects of the expression level of the RFC gene family on six common immune cell infiltration levels: CD4 + T cells, CD8 + T cells, B cells, neutrophils, dendritic cells, and macrophages.

### Availability of data and materials

The datasets used and/or analyzed during the current study are available from the corresponding author on reasonable request.
